# Polygenic Risk Scores in Alzheimer’s Disease: A Cross-Scale View from Genes to Brain and Behavior

**DOI:** 10.3390/biology15201805

**Published:** 2026-10-10

**Authors:** Efthalia Angelopoulou, Ioannis Stamelos, Stefanos N. Sampatakakis, Sokratis Papageorgiou, Nikolaos Scarmeas, Alexandros Hatzimanolis, Christos Proukakis, Chiara Villa

**Affiliations:** 1 1st Department of Neurology, Aiginition Hospital, National and Kapodistrian University of Athens Medical School, 11528 Athens, Greece; angelthal@med.uoa.gr (E.A.); j-stam@hotmail.gr (I.S.); stefanos.sab@gmail.com (S.N.S.); sokpapa@med.uoa.gr (S.P.); ns257@cumc.columbia.edu (N.S.); 2 The Gertrude H. Sergievsky Center, Taub Institute for Research in Alzheimer’s Disease and the Aging Brain, Department of Neurology, Columbia University, New York, NY 10032, USA; 3 1st Department of Psychiatry, Aiginition Hospital, National and Kapodistrian University of Athens Medical School, 11528 Athens, Greece; alhatzi@med.uoa.gr; 4 UCL Queen Square Institute of Neurology, University College London, London WC1N 3BG, UK; c.proukakis@ucl.ac.uk; 5 School of Medicine and Surgery, University of Milano-Bicocca, 20900 Monza, Italy

**Keywords:** Alzheimer’s disease, polygenic risk scores, APOE, amyloid biomarkers, tau pathology, cognitive decline, precision medicine

## Abstract

Alzheimer’s disease (AD) is a genetically complex disorder in which inherited susceptibility is distributed across rare high-impact variants, the apolipoprotein E (*APOE*) locus, and a broad polygenic background of common risk alleles. Polygenic risk scores (PRSs) provide a quantitative framework for summarizing this distributed genetic liability and have become increasingly important tools for risk stratification, cohort enrichment, and biological interpretation. In this review, we synthesize evidence on AD PRSs across multiple biological and clinical scales, moving from genes and molecular pathways to biomarkers, brain structure and function, cognition, behavior, and clinical translation. Genome-wide PRSs capture aggregate inherited risk, whereas pathway-, tissue-, cell-type-, and biomarker-specific PRSs may help clarify the biological routes through which genetic susceptibility is expressed, including amyloid and tau metabolism, lipid homeostasis, endosomal trafficking, immune regulation, glial activation, proteostasis, and mitochondrial quality control. Evidence indicates that AD PRSs are most consistently associated with amyloid-related biomarkers, while associations with tau, neurodegeneration, brain atrophy, functional connectivity, cognitive decline, and neuropsychiatric manifestations are more heterogeneous and often depend on APOE, disease stage, biomarker context, and variable computation methodologies. Although PRSs show promise for research enrichment and mechanistic stratification, their individual-level clinical utility remains limited by modest predictive performance, ancestry-related transferability, calibration challenges, and ethical considerations. Future progress will require multi-ancestry cohorts, integration of common and rare variation, multi-omic annotation, longitudinal modeling, and multimodal prediction frameworks. Ultimately, PRSs are best viewed not as stand-alone diagnostic tools, but as cross-scale instruments for linking inherited susceptibility to the heterogeneous biological and clinical manifestations of AD.

## Simple Summary

Alzheimer’s disease (AD) represents the leading cause of dementia globally and the most common form of neurodegenerative disorder, with a complex genetic etiology. Polygenic risk scores (PRSs), which combine information from multiple genetic variants into a measure of inherited susceptibility, are increasingly used to study AD risk and stratify research cohorts. Here, we discuss PRSs in AD from molecular pathology to brain alterations and ultimately behavioral manifestations. This review highlights the research value of PRSs, paving the way for novel strategies for assessing inherited AD susceptibility.

## 1. Introduction

Alzheimer’s disease (AD) is the leading cause of dementia worldwide and represents one of the most significant public health challenges in the twenty-first century. The Global Burden of Disease Study estimated that 57.4 million people were living with dementia worldwide in 2019 and projected an increase to 152.8 million by 2050 [1]. Despite decades of research, the biological mechanisms underlying AD remain incompletely understood owing to the complex interplay between genetic, environmental, and lifestyle factors.

Genetic susceptibility plays a critical role in the pathogenesis of AD. Twin and family studies have estimated that the heritability of late-onset AD (LOAD) with symptom onset older than 65 years is approximately 58–79% [2], highlighting the substantial contribution of inherited genetic factors to disease risk. Although rare highly penetrant mutations in amyloid precursor protein (*APP*), *PSEN1*, and *PSEN2* cause autosomal dominant forms of early-onset AD (EOAD), the vast majority (more than 95%) of AD cases arise from a complex polygenic architecture involving numerous common and rare variants across the genome, each contributing to the overall disease susceptibility [2].

Among all known genetic risk factors, the ε4 allele of the apolipoprotein E (*APOE*) gene remains the strongest determinant of sporadic AD susceptibility. Individuals carrying one ε4 allele exhibit an approximately threefold increased risk of AD, whereas ε4 homozygotes experience an 8- to 12-fold increased risk and an earlier age at onset [3]. In contrast, the *APOE* ε2 allele exerts a protective effect, reducing AD risk by approximately 40–50% compared with the common ε3 genotype [3]. However, genome-wide association studies (GWAS) have transformed our understanding of the genetic landscape of AD, identifying more than 90 susceptibility loci, including *BIN1*, *CLU*, *PICALM*, *CR1*, *ABCA7*, *TREM2*, and *SORL1* among others [4]. These discoveries have highlighted the involvement of biological pathways implicated in amyloid processing, tau pathology, lipid metabolism, endosomal trafficking, immune regulation, and microglial function [4] in AD pathophysiology, emphasizing that AD is increasingly viewed as the outcome of cumulative genetic perturbations acting across multiple molecular and cellular systems.

The recognition of AD as a polygenic disorder has resulted in the development of polygenic risk scores (PRSs), which aggregate the effects of numerous genetic variants across the genome into a single quantitative measure of inherited susceptibility. PRSs are typically calculated by summing risk alleles weighted according to their effect sizes derived from relevant GWAS, thereby capturing a larger proportion of genetic liability than any individual variant alone. Over the past decade, PRSs have emerged as powerful tools for investigating disease risk, identifying high-risk individuals, stratifying study cohorts, exploring the biological consequences of genetic susceptibility, and subsequently elucidating the biological mechanisms of AD pathogenesis. In addition to pathologically confirmed disease diagnosis of AD [5], PRSs have demonstrated associations with earlier age at onset [6], conversion from mild cognitive impairment (MCI) to dementia [7], as well as longitudinal cognitive decline, often providing predictive information beyond *APOE* genotype alone [8].

Beyond risk prediction, PRSs offer a unique opportunity to investigate how genetic susceptibility manifests across different levels of biological organization. In this context, AD PRSs have been associated with altered amyloid-β (Aβ) and phosphorylated tau levels in cerebrospinal fluid (CSF), increased amyloid and tau deposition in positron emission tomography (PET), structural brain alterations such as hippocampal atrophy and cortical thinning, as well as subtle cognitive differences in cognitively unimpaired individuals [8,9]. These findings suggest that inherited susceptibility summarized by PRSs is associated with measurable differences across the AD continuum, from molecular biomarkers to brain and behavioral phenotypes.

At the same time, recent advances in systems biology have shifted the focus from single-gene associations toward integrated models that consider interactions among genes, molecular pathways, cellular processes, and phenotypic outcomes [2]. Within this framework, PRSs can be conceptualized as biological bridges linking genomic variation to downstream molecular and clinical manifestations [8]. Pathway-specific PRS approaches have further enabled researchers to gain deeper insights into AD biology [8], which could ultimately facilitate the development of precision medicine prevention or treatment strategies.

**Figure 1:**
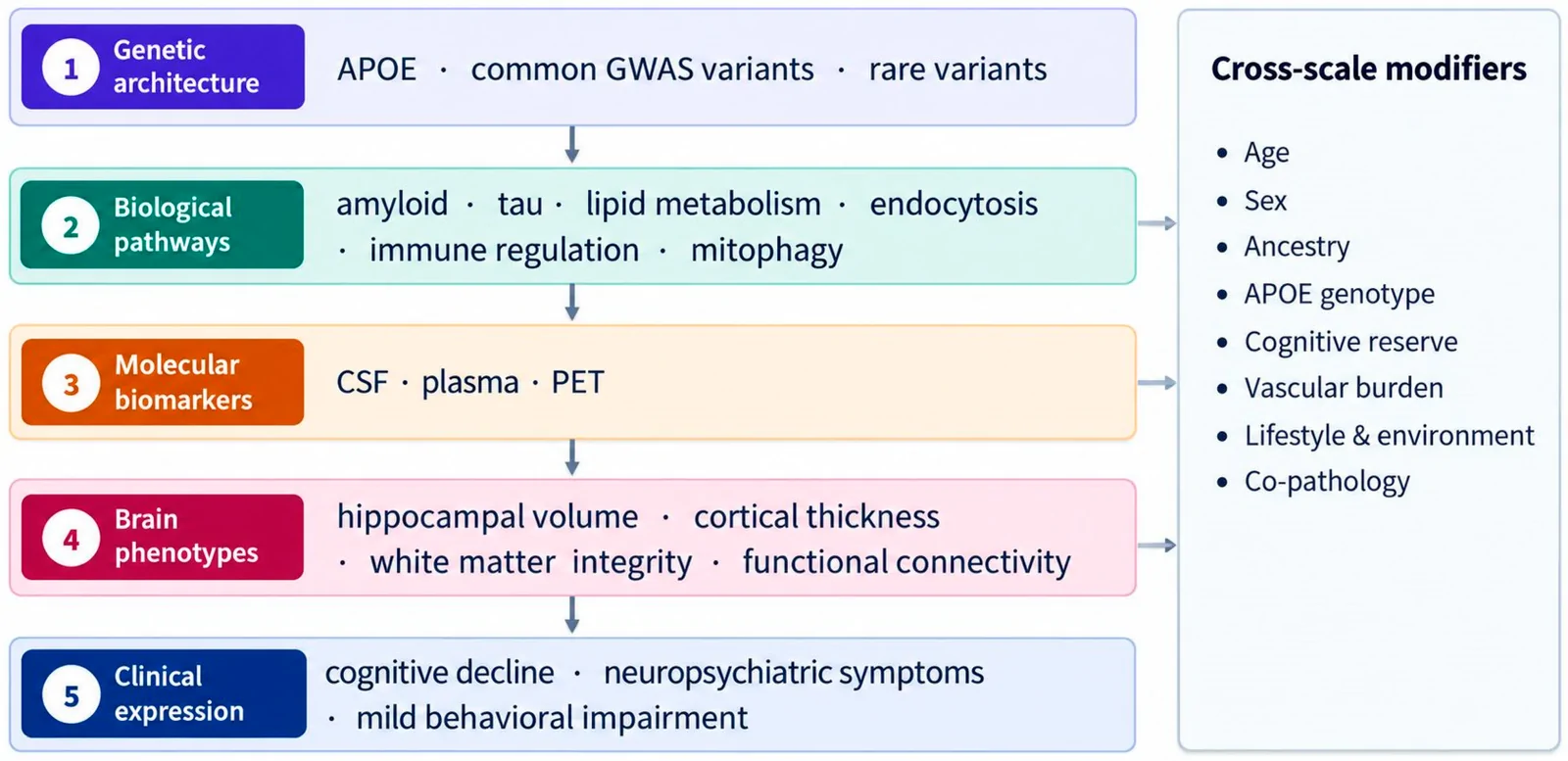
Cross-scale model illustrating how AD polygenic risk may propagate from inherited genetic liability to biological pathways, molecular biomarkers, brain alterations, cognitive and behavioral manifestations, and clinical outcomes.

Earlier reviews have synthesized AD PRS methodology, predictive performance, associations with intermediate phenotypes, and translational challenges [8,9,10]. Building on this work, the present review organizes evidence across genetic architecture, biological pathways, fluid and PET biomarkers, neuroimaging, cognition, and behavior within a common cross-scale framework (Figure 1). Its added value lies in comparing the consistency and limitations of associations across these levels, distinguishing APOE-dependent findings from residual non-APOE associations, and integrating emerging cell-type-specific scores and blood biomarker evidence. This framework is intended to identify supported associations and unresolved mechanistic links, paving the way for novel strategies for assessing AD inherited susceptibility.

For this narrative review, we searched PubMed for relevant original research and review articles published through July 2026. Search terms included AD, dementia, PRSs, polygenic hazard scores and terms relevant to pathways, cell types, biomarkers, neuroimaging, cognition, behavior, and clinical translation. Reference lists of relevant articles were also examined for potential additional reports. Reports were selected for their relevance to AD polygenic risk or hazard scores, their methodological development, or their associations with biological and clinical outcomes. Relevant genetic and functional studies were included to provide mechanistic context. Conference abstracts were considered for emerging findings and were explicitly identified as preliminary evidence. Findings were synthesized narratively across biological and clinical scales. The review was not designed as a systematic review or meta-analysis. The last search date was 30 July 2026.

## 2. PRS in AD: Concepts and Methodological Foundations

### 2.1. Genetic Architecture of AD

**Figure 2:**
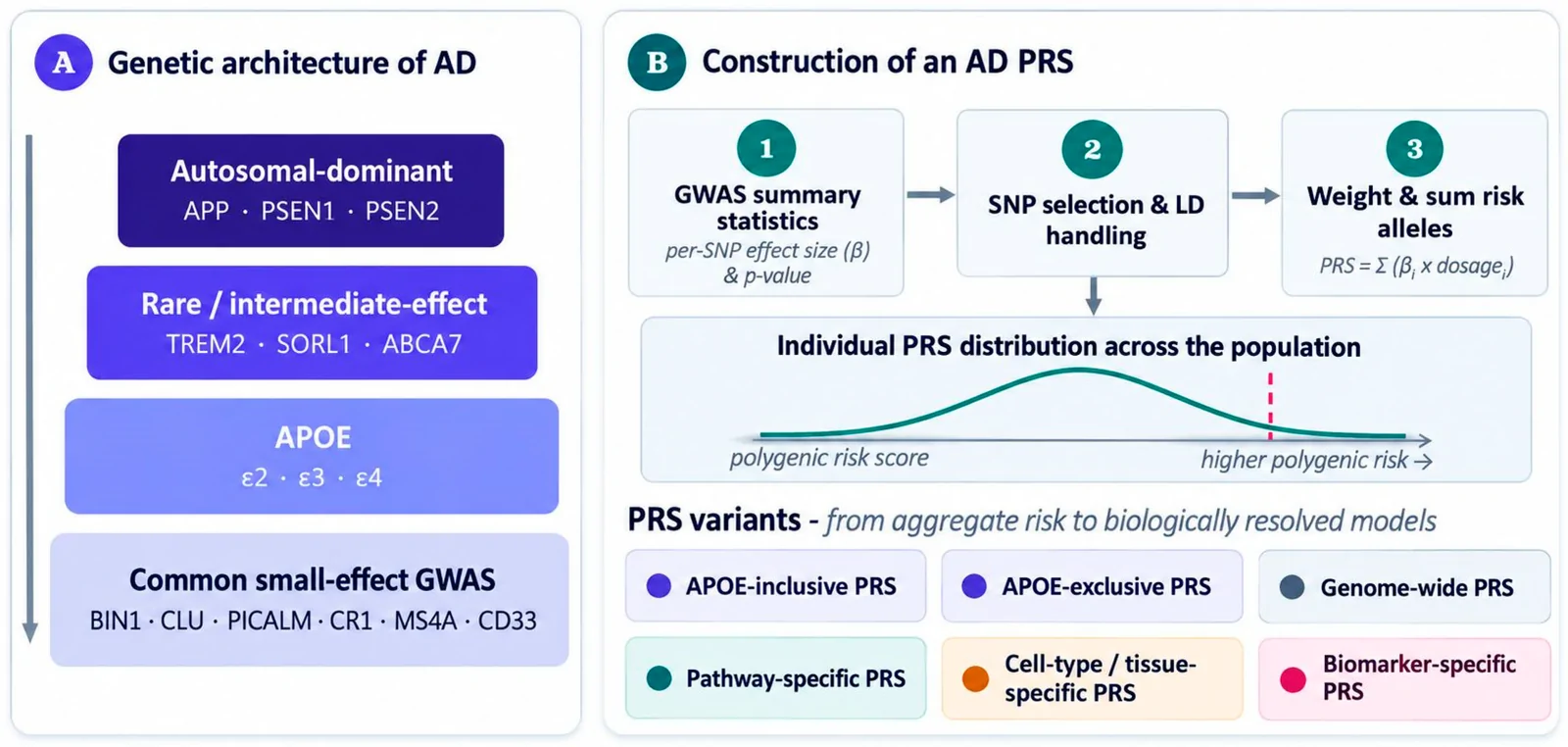
Genetic architecture of AD and methodological principles of PRS construction, highlighting the distinction between aggregate risk prediction and biologically resolved PRS models.

LOAD is characterized by a layered genetic architecture, in which a small number of high-impact genetic factors coexist with a broad background of common and rare variants, which collectively affect disease susceptibility. This architecture is particularly well suited to polygenic modeling, as the genetic contribution to AD risk is distributed across numerous loci of individually relatively limited predictive value (Figure 2).

The strongest genetic determinant of sporadic AD remains the *APOE* locus, whose ε2, ε3 and ε4 alleles exert isoform-specific effects on disease susceptibility, amyloid deposition, lipid transport, neuroinflammation and vascular integrity [11]. However, the genetic landscape of AD extends far beyond *APOE*. GWAS have progressively identified an increasing set of susceptibility loci, many of which map onto convergent biological pathways rather than individual gene effects. Large-scale studies established some key non-*APOE* loci, including *BIN1*, *CLU*, *PICALM*, *CR1*, *ABCA7*, *MS4A*, *CD33*, *EPHA1*, *SORL1*, and *TREM2*, implicating lipid metabolism, immune regulation, endocytosis, complement activation, and microglial biology in AD pathogenesis [4,12,13,14].

The number of reproducible AD-associated loci has increased significantly with advances in sample size, imputation methods, and incorporation of clinically diagnosed, proxy and biobank-based phenotypes. Kunkle and colleagues analyzed 94,437 individuals and confirmed 20 previously reported loci, while identifying 5 novel genome-wide significant risk loci, further strengthening the involvement of APP metabolism, tau binding, immunity, and lipid processing pathways [13]. Jansen and colleagues, incorporating clinically diagnosed AD and AD-by-proxy phenotypes, analyzed 71,880 cases and 383,378 controls and identified 29 risk loci, with gene prioritization implicating 215 potential causal genes and enrichment in microglial and immune-related tissues [14]. Bellenguez and colleagues subsequently performed a two-stage GWAS including 111,326 clinically diagnosed or proxy AD cases and 677,663 controls and reported 75 risk loci, of which 42 were novel [15]. Most recently, a 2026 consensus meta-analysis of European-ancestry GWAS comprising 128,681 AD and AD-related dementias (ADRD) cases or proxy cases and 849,833 controls or proxy controls identified 91 genome-wide significant loci associated with AD/ADRD risk, including 16 novel ones. Importantly, 56 of these loci were specifically detected in clinically diagnosed AD cases [4].

However, these figures should be interpreted with some caution. The number of AD risk loci depends on phenotype definition, ancestry, inclusion of proxy cases, imputation methods, statistical threshold, and whether loci requiring external validation are counted. Therefore, based on currently established AD/ADRD GWAS evidence, the number of genome-wide significant risk loci is best described as exceeding 90 [4]. This distinction is important for PRS construction, because conventional AD PRSs may include either genome-wide significant variants or a broader set of predominantly common variants, including those that do not reach genome-wide significance.

The effect-size distribution of AD risk variants further explains why PRSs are necessary. With the exception of *APOE*, most common-variant GWAS loci confer small increments in risk, often with odds ratios in the range of approximately 1.05–1.30 [15]. In contrast, selected rare variants may exert larger effects. For example, rare variants in *TREM2*, *SORL1*, and *ABCA7* may confer moderate-to-large risk effects, although their low frequency limits their contribution to population-level prediction [16]. Coding variants in *TREM2* illustrate the intermediate-effect component of AD genetic architecture: R47H has been associated with an approximately 3.9-fold increased risk of AD, whereas R62H appears to confer a more modest effect [17]. More recent rare-variant syntheses suggest that at least 13 genes influence AD risk through rare variants and estimate that approximately one in seven individuals with late-onset AD may carry a rare genetic risk variant [16].

Thus, AD genetic architecture is best understood as a continuum rather than a dichotomy between monogenic and polygenic disease. At one end of the genetic architecture are rare autosomal dominant mutations in *APP*, *PSEN1*, and *PSEN2*, which account for a small minority of AD cases and are primarily associated with early-onset familial disease. At the other end lies the highly polygenic background of LOAD, shaped by thousands of common variants of small effect. Between these extremes lie rare and low-frequency variants of intermediate effect, particularly in genes related to microglial activation, lipid handling, endosomal trafficking, and amyloid processing. PRSs are designed to quantify the cumulative burden of this distributed genetic liability and therefore provide a framework for linking inherited risk to downstream phenotypes.

### 2.2. Construction of PRS for AD

The construction of a PRS for AD begins with the translation of GWAS summary statistics into an estimate of genetic susceptibility at an individual level [18]. PRSs in their simplest form can be calculated as the weighted sum of risk alleles carried by an individual, with the weight of each allele representing the corresponding effect size estimated in an independent GWAS. For binary traits such as AD, these weights are usually derived from the logarithm of the odds ratio for each variant, and the score positions an individual along a continuous distribution of genetic susceptibility within a given population [18]. PRS can be simply expressed as:

PRS = Σ β_i_G_i_

where β_i_ denotes the GWAS-derived effect size for variant i, and G_i_ represents the number of risk alleles carried by the individual at this specific locus, usually coded as 0, 1, or 2 [18]. As raw PRS values depend on the number of included variants, the allele-coding scheme, the discovery GWAS and the ancestry composition, PRSs are usually standardized within the target sample to facilitate interpretation [18].

Variant selection is a key methodological step in PRS construction. Whereas early AD scores often relied on genome-wide significant loci (*p* < 5 × 10^−8^), contemporary PRSs typically include subthreshold variants across multiple significance thresholds to better capture polygenic liability [9]. The most widely used strategy is clumping and thresholding, in which variants are pruned for linkage disequilibrium and then selected across predefined GWAS *p*-value thresholds, ranging from stringent cutoffs such as 5 × 10^−8^ or 1 × 10^−5^ to more permissive inclusion thresholds such as 0.05, 0.1, 0.5, or 1.0 [9]. The optimal threshold is typically determined empirically, as AD risk reflects both the large effect of APOE and a diffuse background of small-effect variants. LD-aware Bayesian methods provide an alternative to clumping and thresholding. Both LDpred and PRS-CS estimate posterior single nucleotide polymorphism (SNP) effects from GWAS summary statistics and an LD reference panel, but differ in their prior assumptions: the original LDpred model uses a point-normal prior, whereas PRS-CS uses continuous shrinkage priors [19]. Their accuracy, however, remains dependent on discovery GWAS size and quality, imputation accuracy, ancestry-matched LD reference panels, and genetic similarity between discovery and target populations.

Handling the *APOE* region is another key issue in AD PRS construction. As *APOE* ε4 can dominate prediction, studies often compute both *APOE*-inclusive and *APOE*-exclusive PRSs, to distinguish total genetic risk from residual non-*APOE* polygenic burden [9].

While genome-wide PRSs prioritize predictive performance over mechanistic specificity, pathway-specific PRSs include only loci mapped to predefined biological pathways or gene sets, such as amyloid processing, tau-related mechanisms, lipid and cholesterol metabolism, endocytosis, immune signaling, and microglial function [9,20,21]. These scores can help elucidate whether specific biological pathways are linked to AD endophenotypes related to specific biomarkers, brain changes, cognitive decline, or disease progression. Pathway-specific PRSs require careful definition of gene sets and variant-to-gene mapping [22]. Gene sets may be derived from curated pathway databases, expert annotations, gene-expression profiles, protein-interaction networks, or cell-type-specific data, while variants are typically assigned to genes using proximity, expression quantitative trait loci (eQTL), chromatin-interaction, or functional annotation [22]. These choices influence biological interpretation, particularly in AD, where genetic effects may act through multiple cell types, including microglia, astrocytes, neurons, vascular cells, and peripheral immune mechanisms.

Beyond conventional PRSs, time-to-event-specific approaches include polygenic hazard scores, which incorporate age-specific disease incidence to estimate age-dependent AD risk [6]. Multimodal models further combine PRSs with biomarkers, neuroimaging, family history, vascular factors, or cognition to improve risk stratification.

Overall, AD PRS construction depends on several methodological choices, including the discovery GWAS, LD handling, SNP inclusion strategy, treatment of APOE, ancestry matching, and use of genome-wide versus pathway-specific scores. These decisions shape both predictive accuracy and biological interpretation, making PRSs valuable not only for risk stratification but also for linking inherited susceptibility to molecular, brain, and cognitive phenotypes.

As a historical quantitative benchmark, the systematic review by Harrison and colleagues reported areas under the curve (AUCs) of 0.62–0.84 in studies including APOE and 0.57–0.75 in studies excluding APOE for AD case–control discrimination [8]. However, as these AUCs are descriptive ranges across heterogeneous studies, they do not reflect pooled estimates and they cannot be used for prediction of MCI progression, biomarker positivity or individual lifetime risk.

Table 1 summarizes selected AD polygenic risk and hazard score studies, including their discovery datasets, target samples, APOE handling, variant-selection strategies, and reported performance or association measures. However, as study populations, outcomes, covariates, and validation procedures differ between these studies, these estimates are not directly comparable. AUC quantifies discrimination, whereas hazard ratios quantify relative associations and incremental R^2^ describes improvement in model fit. Discovery-target overlap and selection of score thresholds within the evaluation sample can produce optimistic estimates. Independent evaluation is therefore required to establish the incremental predictive value of PRSs beyond established clinical factors and APOE.

Sample sizes refer to the reported study datasets; individual analyses may use smaller outcome-specific subsets. AUC measures discrimination, HR quantifies relative hazard, and ΔR^2^ describes incremental model fit. These measures are not directly interchangeable. Results should be interpreted in relation to the outcome, covariate adjustment, APOE handling, and validation design of each study. In a study by Pyun et al., scores were standardized using an amyloid-negative cognitively unimpaired reference group [23].

### 2.3. Current Methodological Challenges

Despite substantial progress, there are several methodological challenges that may affect the interpretation, generalizability, and clinical translation of PRSs in AD. Within a cross-scale framework, biases related to genetic risk estimation become even more important, since these limitations may propagate downstream to analyses of biomarkers, neuroimaging phenotypes, and clinical outcomes.

First, population stratification and limited cross-ancestry portability represent related but distinct challenges. Adjustment for genetic principal components can reduce confounding by population structure, but does not by itself resolve differences in variant frequencies, LD patterns, tagging of causal variants, or the precision of effect estimates across discovery and target populations. The frequently cited several-fold differences in PRS accuracy derive from broader cross-trait evidence, and they should not be interpreted as a uniform reduction established for all AD scores or populations [25]. In AD, recent evidence indicates that European-derived polygenic scores retain associations with disease risk in several populations of diverse ancestries, while cross-ancestry scores can strengthen these associations, particularly when the APOE region is included [9,26]. Although these findings support partially shared genetic susceptibility, they cannot establish equivalent predictive performance or calibration across populations.

Population-specific discovery studies also reveal susceptibility signals that may be poorly captured by European-derived scores. For example, an African-ancestry GWAS identified a genome-wide significant association at *MPDZ* on chromosome 9p23 (rs141610415) [27]. However, detection in an African-ancestry sample does not prove that the locus acts exclusively in that population, or that its inclusion would definitely improve PRS performance. These findings highlight the importance of diverse discovery datasets and population-specific evaluation of PRS performance [26,27].

A second issue concerns absolute risk calibration and age-dependent clinical expression of genetic susceptibility. A PRS percentile indicates an individual’s position within a reference distribution, but it is not the probability of developing AD. Estimating absolute risk requires a model specifying the outcome, the prediction horizon, and the target population, with an appropriate baseline incidence or hazard and relevant clinical predictors [28]. Calibration should be evaluated by comparing predicted probabilities with observed outcomes over the same period in independent, representative data. Good discrimination does not guarantee accurate absolute risk estimates. For a fixed score definition, although the underlying genetic score remains stable, the probability of clinical disease may change with age or clinical context. Thus, age-dependent penetrance should be understood as the probability of clinical expression by a specified age. Estimates of remaining lifetime risk should also account for the competing risk of death. Nevertheless, age-related risk modeling, as illustrated by polygenic hazard scores [6], does not remove the need for external validation and population-specific calibration.

A third limitation is heterogeneity in discovery phenotypes. Large GWAS combine varying proportions of clinically diagnosed AD, AD-by-proxy, and broader AD/ADRD definitions [5,14,15]. Proxy phenotypes increase sample size but infer disease liability from relatives’ reported diagnoses, which may be affected by diagnostic uncertainty and relatives’ age and survival. Broader dementia definitions can additionally include mixed or non-AD pathologies. Consequently, the resulting SNP weights may capture susceptibility to a heterogeneous clinical dementia phenotype rather than exclusively to biomarker-defined AD. This may attenuate or complicate associations with specific downstream outcomes, including tau pathology and neurodegeneration, which themselves differ in disease specificity. Such heterogeneity may be a plausible contributor to inconsistent PRS-biomarker findings. Where available, comparisons using clinically diagnosed-only or no-proxy discovery GWAS can help assess robustness.

Furthermore, there are significant methodological discrepancies across relevant studies, including differences in the discovery GWAS, imputation panel, LD reference, SNP inclusion threshold, Bayesian shrinkage method, treatment of the *APOE* region, and covariate adjustment. As a result, two studies, for example, may both report an “AD PRS” while in fact using scores with different variant composition, biological meaning, and predictive behavior. This lack of standardization may obscure whether inconsistent findings reflect true biological differences or methodological variations.

Finally, PRSs should not be interpreted as deterministic predictors, since several factors, including education, cardiovascular profile, and physical activity, among others affect AD risk. The greatest value of PRSs rather lies in combination with molecular and neuroimaging biomarkers, along with lifestyle and longitudinal clinical data, where they can help clarify how inherited susceptibility may modify disease biology across the AD continuum.

## 3. From Genes to Biology: Pathways Captured by AD PRSs

**Figure 3:**
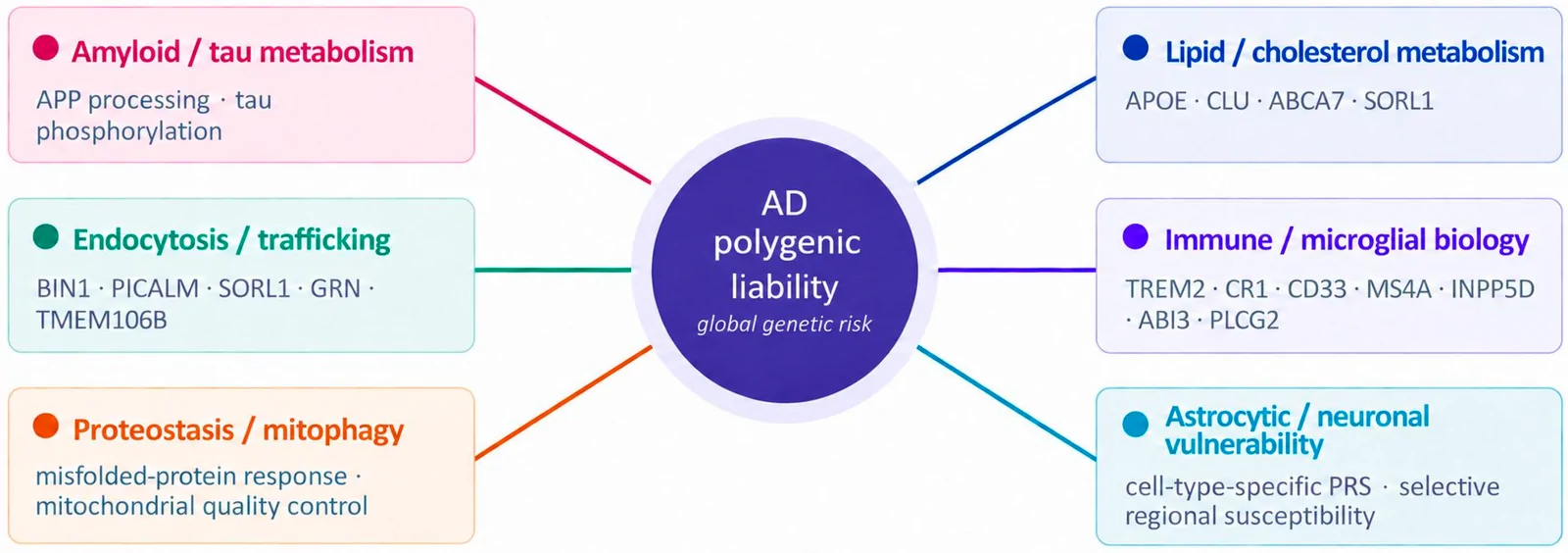
Biological themes investigated using pathway- and cell-type-specific AD PRSs. These approaches may help relate aggregate genetic susceptibility to amyloid and tau metabolism, lipid homeostasis, trafficking, immune processes, proteostasis, and cellular vulnerability. The schematic summarizes biological hypotheses and proposed associations.

While genome-wide PRSs aggregate multiple variants into a single susceptibility measure, pathway-specific PRSs aim to decompose this aggregate into components with biological interpretability, including amyloid and tau metabolism, lipid and cholesterol homeostasis, endocytosis, immune regulation, microglial function, protein homeostasis, and mitochondrial quality control (Figure 3).

### 3.1. Amyloid and Tau-Related Pathways

EOAD and LOAD are characterized by the accumulation of both extracellular Aβ plaques and intracellular tau-containing neurofibrillary tangles (NFT) in the brain [29]. While in autosomal dominant AD, *APP*, *PSEN1*, and *PSEN2* gene mutations directly affect amyloid processing, in LOAD, common genetic risk seems to modulate amyloid and tau pathology through broader indirect systems, including endosomal trafficking, lipid transport, immune clearance, and protein quality control. In particular, GWAS pathway analyses have identified amyloid- and tau-related biological processes among the most significantly enriched gene sets, supporting their central contribution to AD pathophysiology [15]. Importantly, genetic risk loci have been related to deregulation of both amyloidogenic and non-amyloidogenic pathways implicated in APP metabolism, suggesting that polygenic susceptibility may alter the balance between Aβ-generating and Aβ-sparing APP processing routes [15].

Furthermore, GWAS pathway-enrichment and single-cell expression analyses have shown that several prioritized AD-associated genes, including *RHOH*, *BLNK*, *SIGLEC11* and *LILRB2*, were predominantly expressed in microglia, with some linked to Aβ peptides or amyloid plaques [15]. Thus, amyloid- and tau-related genetic influences may extend beyond neuronal APP processing and protein aggregation to include microglial mechanisms of plaque recognition, containment, and clearance.

### 3.2. Lipid and Cholesterol Homeostasis

Lipid and cholesterol metabolism are among the most reproducible domains in AD genetics, linking also molecular and cellular levels of AD pathogenesis. Beyond *APOE*, loci such as *CLU*, *ABCA7*, and *SORL1* link lipid handling to amyloid clearance, membrane trafficking, phagocytosis, synaptic resilience, glial activation, myelin integrity, and vascular vulnerability [3,15]. Astrocytes and microglia are major sources of APOE in the brain; ABCA7 influences lipid transport and phagocytic function; CLU participates in lipid transport and extracellular chaperone activity; and SORL1 links trafficking biology with APP sorting [15]. Pathway-specific PRSs may therefore help clarify how lipid-related inherited risk contributes to AD pathophysiology and biomarkers.

### 3.3. Endocytosis and Vesicular Trafficking

Endocytosis and vesicular trafficking are key pathophysiological mechanisms in AD, as APP processing occurs partly within endosomal compartments, while amyloid clearance, tau propagation, and microglial phagocytosis also depend on vesicular trafficking systems. An APOE-excluded PRS related to endocytosis and fibril-formation has been associated with amyloid positivity in the Alzheimer’s disease neuroimaging initiative (ADNI), supporting a genetic link between endocytosis and amyloid pathology in AD [21].

Some AD-associated loci implicate endosomal-lysosomal biology that is also relevant to other neurodegenerative disorders. GRN and TMEM106B illustrate this overlap with frontotemporal degeneration [15,30]. For Parkinson’s disease (PD), *TMEM106B* genotype has been associated with longitudinal cognitive decline, although further evidence is needed to clarify if cognitive impairment in PD and AD shares a common polygenic component.

### 3.4. Immune Regulation, Complement Activation, and Microglial Biology

One of the major achievements of AD GWAS has been the shift from a neuron-centric view of genetic risk to a multicellular model in which innate immunity and microglial biology are central. Loci such as *CR1*, *CD33*, *MS4A*, *INPP5D*, *ABI3*, *PLCG2*, and *TREM2* implicate complement activation, phagocytosis, immune signaling, and microglial response states [14]. Jansen and colleagues demonstrated that AD-prioritized genes were enriched in immune-related tissues and cell types, including microglia, while accumulating additional evidence further supporting the implication of neuroinflammation in AD pathophysiology [14].

Pathway PRSs add a further layer by linking immune-related genetic burden to AD endophenotypes. Darst et al. included immune-response scores in their preclinical pathway PRS framework [20], while Schork and Elman identified immune signaling, inflammatory response including microglial activation, and humoral immune response including complement activation among AD-associated pathway clusters [21].

Recent GWAS in AD have further prioritized several genes related to the linear ubiquitin chain assembly complex (LUBAC), including *SHARPIN*, *RBCK1*, and *OTULIN*, highlighting a potential role for linear ubiquitination in AD [15]. LUBAC regulates innate immune signaling, NLRP3 inflammasome activation, autophagy, and TNF-α signaling [15,31]. These processes have been experimentally linked to Aβ accumulation, tau pathology, and synaptic dysfunction [31], suggesting that AD inflammatory genetic risk may link immune activation with Aβ pathology, tau pathology, synaptic dysfunction, and broader intercellular crosstalk. Notably, phosphorylated tau positivity in ADNI was associated with an immune-signaling PRS [21], reinforcing the notion that immune-related inherited risk may influence tau-associated disease biology, not only amyloid clearance.

### 3.5. Protein Homeostasis, Misfolded-Protein Response, and Mitophagy

Recent pathway PRS findings also highlight mechanisms that may be more closely related to downstream neurodegeneration than to early amyloid deposition. In the ADNI study, both phosphorylated tau positivity and hippocampal volume were related to protein localization and mitophagy PRSs [21]. As hippocampal atrophy reflects neuronal and synaptic injury rather than amyloid deposition alone [32], these findings suggest that genetic burden in pathways related to protein trafficking, mitochondrial quality control, and proteostasis may influence cellular vulnerability after, or in parallel with, amyloid and tau accumulation [33]. Hence, pathway PRSs related to protein localization and mitophagy might capture genetic susceptibility to cellular stress, mitochondrial dysfunction, or impaired proteostasis. Within the genes-to-brain-to-behavior framework, such pathways may help explain why individuals with comparable amyloid or tau burden can differ in the extent of brain atrophy and cognitive impairment.

Comparative mammalian studies provide additional biological context for mitochondrial quality control. Analyses of buffalo cerebellum relative to peripheral tissues identified expression patterns involving regulated mitochondrial transport, metabolic signaling, and oxidative protein repair, including MSRA and MSRB2 [34]. A complementary study highlighted mitochondrial gene-expression maintenance, including PNPT1-associated RNA surveillance, and reported conservation of selected regulatory expression patterns between buffalo and human reference datasets [35]. These observations are consistent with a proposed ”precision-over-throughput” framework, emphasizing maintenance and repair alongside energy production. Such a framework offers a rationale for investigating whether distributed genetic variation in mitochondrial maintenance pathways contributes to neural vulnerability. Nevertheless, the relationship between AD PRS and mitochondrial failure or neuronal cell death remains to be elucidated in future studies.

### 3.6. Interpretation and Limitations

Pathway-specific PRSs are best understood as tools for biological interpretation rather than accurate predictors. The Darst et al. study showed that APOE-inclusive pathway scores were largely driven by APOE [20], while the Schork and Elman ADNI analysis found that a global AD PRS outperformed pathway PRSs for associations with diagnosis and biomarkers [21]. These findings indicate that genome-wide PRSs remain better suited for aggregate risk stratification, whereas pathway PRSs are more useful for decomposing genetic liability into mechanistic components.

At the same time, pathway-specific PRSs retain several of the limitations inherent to conventional PRS methods. Pathway scores depend on gene-set definitions, variant-to-gene mapping, pathway databases, and cell-type annotations. Biological pathways also overlap substantially, as lipid metabolism affects amyloid clearance, endocytosis affects APP processing, immune activation affects plaque biology, and mitophagy contributes to neurodegeneration. Therefore, the value of pathway PRSs mainly lies in understanding through which molecular systems the genetic risk for AD may be expressed. This would be the first step in a cross-scale model of AD, linking genes to molecular pathways, biomarkers, brain alterations, and finally cognitive outcomes.

Interpretation of residual non-APOE burden requires distinguishing three questions: whether the *APOE* region was excluded from score construction, whether an association persists after adjustment for *APOE* genotype, and whether the score improves prediction beyond APOE and established clinical predictors. These are different levels of evidence. Exclusion boundaries should be reported because removing only the *APOE* ε2/ε4-defining variants may leave correlated regional information within the score. Moreover, independence from APOE does not imply independence from amyloid pathology. Evidence of incremental predictive value requires evaluation of models with and without the non-APOE score in independent data.

Sex is another potential modifier of the relationship between polygenic susceptibility and clinical expression. A study using sex-stratified genetic discovery and independent validation reported sex-dependent autosomal polygenic associations with AD onset and progression after accounting for APOE [36]. Future analyses should distinguish adjustment for sex from formal testing of PRS-by-sex interactions and evaluate whether predictive performance and calibration differ by sex.

## 4. From Biology to Biomarkers: PRS Associations with AD Biomarkers

Biomarkers provide the first measurable intermediate layer between inherited genetic risk and clinically expressed AD. Within the A/T/N framework, amyloid biomarkers reflect Aβ plaque pathology, tau biomarkers reflect phosphorylated tau and NFT-related processes, and neurodegeneration markers capture downstream neuronal or synaptic injury. For this reason, PRS-biomarker association studies may help us understand whether genetic contributions are already detectable at the level of molecular pathology before its clinical expression.

Existing evidence suggests that AD PRSs are most consistently associated with amyloid-related biomarkers, particularly when the *APOE* region is included [8]. On the other hand, associations with tau and neurodegeneration markers are relatively more heterogeneous [8]. Emerging pathway- and cell-type-specific PRS approaches suggest that genetic risk for AD may influence different biomarkers through at least partially distinct biological mechanisms [37]. In this regard, biomarker-specific PRS analyses can help identify whether inherited risk can be preferentially expressed through amyloid accumulation, tau dysregulation, glial activation, or downstream neurodegeneration, providing further insights into how polygenic susceptibility is translated into specific molecular stages of the AD continuum.

### 4.1. CSF Biomarkers: Amyloid-β, Total Tau, and Phosphorylated Tau

Early PRS-biomarker studies focused mainly on CSF, with CSF Aβ42, total tau, and phosphorylated tau providing direct biochemical readouts of AD-related pathology. In an early clinical and neuropathological study by Martiskainen and colleagues, a PRS for AD containing 22 loci was associated with lower CSF Aβ42 levels in the clinical cohort and altered soluble Aβ42 levels and γ-secretase activity in the temporal cortex tissue [38]. The association with γ-secretase activity was independent of *APOE* [38], suggesting that non-APOE polygenic AD burden may influence APP processing and Aβ metabolism. In another study by Sleegers and colleagues using a 22-SNP PRS for AD, higher PRS was associated with earlier onset age and lower CSF Aβ42 levels, whereas no significant associations were reported for total tau or pTau181 [39]. Extending these observations, a polygenic hazard score study in ADNI showed that higher genetic hazard for AD was associated with increased likelihood of amyloid and total tau CSF positivity among cognitively normal and MCI participants, supporting the use of polygenic measures as enrichment markers for preclinical AD biomarker abnormalities [40]. These early findings support a recurring pattern, where PRSs for AD most robustly track amyloid-related CSF changes, while tau might require more specific genetic models or analyses stratified by disease stage.

More recent studies have distinguished amyloid-dependent from amyloid-independent genetic effects on tau. In the Swedish BioFINDER study, APOE-excluded AD PRSs were associated with CSF tau biomarkers, with selected associations persisting after adjustment for Aβ measures [41]. These findings support residual associations beyond measured amyloid pathology, although they cannot establish an amyloid-independent causal mechanism. In the population-based Gothenburg H70 Birth Cohort, a non-APOE AD PRS was associated with CSF Aβ42 and neurofilament light (NfL), suggesting that genes increasing AD risk may also be involved in neuroaxonal injury [42].

A particularly important development has been the emergence of cell-weighted PRSs. In BioFINDER-1, Kumar and colleagues generated six cell-weighted AD PRSs using single-cell RNA sequencing annotations for astrocytes, excitatory neurons, inhibitory neurons, microglia, oligodendrocyte precursor cells, and oligodendrocytes, alongside a full non-cell-specific PRS [37]. Neuronal PRSs showed strong associations with CSF Aβ, pTau217 and cognition, whereas microglial PRSs showed stronger associations with CSF pTau217 than Aβ [37]. Moreover, Aβ positivity only partly mediated PRS-pTau217 associations, with the lowest mediation observed for microglial PRSs [37]. These findings suggest that microglial genetic liability may contribute to soluble phosphorylated tau biology partly independently of amyloid burden. In the same vein, another study showed that astrocyte activation PRS was linked to CSF Aβ42 levels, and amyloid burden modified the relationship between this PRS and CSF phosphorylated tau levels [43]. These results suggest that cell-type-informed PRSs may clarify how AD genetic risk is expressed through distinct neuronal and glial mechanisms, with amyloid pathology potentially interacting with microglial and astrocytic genetic liability to influence downstream tau-related biomarker changes.

Interestingly, a liver-specific AD PRS was also associated with AD diagnosis and CSF Aβ42/Aβ40 and pTau/Aβ42 ratios [44]. Although these CSF associations were partly dependent on inclusion of the APOE region and require replication in larger biomarker cohorts, they suggest that functionally annotated PRSs may help link systemic metabolic or lipid-related genetic biology with central AD biomarker profiles.

Taken together, CSF studies suggest that conventional AD PRSs are most consistently associated with amyloid-related changes, particularly reduced Aβ42, whereas tau associations become clearer when more targeted genetic models are used, such as amyloid-adjusted, cell-weighted, or potentially pathway-specific PRS. This suggests that amyloid and tau biomarker abnormalities may reflect partly overlapping but biologically distinguishable expressions of inherited AD liability. They also suggest that PRSs may also reflect broader neurodegeneration-related processes such as neuroaxonal injury. In this framework, CSF biomarkers provide an important intermediate layer between genetic susceptibility and downstream brain changes, helping to clarify whether polygenic risk is expressed primarily through amyloid metabolism, tau dysregulation, or cell-type-specific mechanisms involving neurons and microglia.

### 4.2. Amyloid PET: Genetic Risk and In Vivo Aβ Accumulation

Compared with CSF biomarkers that capture earlier and more dynamic changes in soluble Aβ metabolism, amyloid PET provides an in vivo measure of established Aβ plaque burden and deposition. In the Flemish Prevent AD Cohort KU Leuven, Luckett and colleagues demonstrated that PET amyloid accumulation in older adults with normal cognition was most clearly associated with a combined genetic score incorporating both APOE and non-APOE PRSs, whereas non-APOE PRSs showed limited associations [45]. Similarly, in the Wisconsin Registry for Alzheimer’s Prevention, Darst and colleagues demonstrated that pathway-specific PRSs for Aβ clearance, cholesterol metabolism, and immune response were linked to preclinical amyloid deposition, although these associations were mainly driven by APOE [20]. Together, these studies support a prominent contribution of APOE to the observed associations with amyloid PET measures, although they do not, by themselves, establish independent non-APOE pathway contributions to amyloid positivity.

### 4.3. Tau PET and Tau-Focused Genetic Risk

Tau biomarkers are generally more closely related to neurodegeneration and cognitive decline compared to amyloid biomarkers alone, motivating the development of tau-focused PRSs derived from tau PET or tau-related endophenotypes [46]. A key example is the tau PRS study using multimodal data to derive a PRS for tau PET deposition, in which a tau-specific PRS was hypothesized to be able to capture inherited susceptibility to tau accumulation beyond amyloid burden, *APOE* ε4, and conventional non-APOE AD PRSs. The tau PRS was strongly associated with tau PET deposition, and, in independent validation cohorts, it was associated with CSF phosphorylated tau and postmortem Braak NFT stage [46]. These findings show that biomarker-derived PRSs can capture genetic architecture specific to a pathological process, rather than relying only on clinical AD diagnosis. Hence, tau-specific PRS models might be particularly useful for understanding why individuals with similar amyloid burden can differ markedly in tau accumulation and clinical progression, paving the way for biomarker-specific genetic stratification and more precise models of disease heterogeneity.

### 4.4. Blood-Based Biomarkers: Emerging but Still Limited PRS Evidence

Blood-based biomarkers, including plasma Aβ42/Aβ40, pTau217, pTau181, glial fibrillary acidic protein (GFAP), and NfL, are transforming AD research by providing scalable measures of amyloid pathology, tau pathology, astroglial activation, and neuroaxonal injury [47]. Plasma pTau217 shows particularly strong performance for biologically defined AD, while Aβ42/Aβ40 and pTau/Aβ ratios may improve detection of amyloid-related pathology and support risk stratification across the AD continuum [47]. GFAP may reflect astroglial activation and NfL provides a less AD-specific marker of neuroaxonal injury.

Evidence linking AD PRSs to blood-based biomarkers remains limited. A conference abstract reported that a microglia-specific AD PRS was associated with longitudinal increases in plasma pTau217 and faster cognitive decline in cognitively unimpaired older adults [48]. The authors reported an estimated mediation proportion of approximately 42% through increasing pTau217. However, this estimate does not establish causal mediation, and the methodological details are not available; hence, this preliminary finding requires replication in future studies.

Another recent line of evidence comes from GWAS of plasma AD biomarkers themselves. In the Korean K-ROAD cohort, GWAS of plasma pTau217, pTau181, NfL, and GFAP identified genome-wide significant loci for each biomarker, including strong associations at the *APOE* locus and biomarker-specific signals such as DACT1-DAAM1 for pTau217, KCNJ3 for pTau181, KLHDC4 for NfL, and SLC10A7 for GFAP [49]. These findings suggest that blood-based AD biomarkers might have partly distinct genetic underpinnings. Future models may therefore integrate disease risk PRSs with biomarker-specific genetic scores to better capture the molecular regulation of circulating tau, glial, and neurodegeneration markers.

Although circulating concentrations are not direct readouts of CNS polygenic liability, the scalability of blood-based biomarkers makes their integration with PRSs attractive for research stratification. They may reflect biomarker release, transport into blood, distribution volume, and systemic clearance, alongside the underlying cerebral pathology. Lower estimated glomerular filtration rate (eGFR), a marker of reduced kidney function, has been associated with higher plasma pTau181 and pTau217 concentrations, thereby complicating interpretation. The pT217/T217 ratio, calculated using phosphorylated and corresponding unphosphorylated tau peptides, has shown weaker associations with renal dysfunction than pTau217 concentration alone in the studied cohorts [50]. Nevertheless, kidney function may affect tau ratios in yet unclear ways, and future real-world evidence is needed to clarify this relationship.

Body mass index and vascular comorbidities introduce additional complexity. Higher BMI has been associated with lower blood NfL concentrations, with blood-volume-related dilution proposed as one explanation. Nevertheless, BMI-related associations should be evaluated separately for each biomarker [47]. Vascular disease may influence biomarker levels through coexisting brain injury and systemic illness. In particular, GFAP and NfL reflect astroglial and neuroaxonal responses that are not specific to AD, so their concentrations may capture vascular or other neurological injury alongside AD-related processes [47]. These factors may therefore contribute to variability in PRS-biomarker associations, while direct evidence that they explain the heterogeneity of those associations remains limited.

Analytical differences also require careful consideration. Plasma pTau217 assays differ in measurement technology, including immunoassay and mass spectrometry, and in whether they quantify phosphorylated tau concentration or its ratio to the corresponding unphosphorylated peptide. Head-to-head comparisons demonstrate that tests targeting the same phosphorylation site can differ in their associations with AD pathology and diagnostic performance, suggesting that concentrations and cutoffs may change substantially across platforms [51]. PRS-plasma studies should report assay and preanalytical procedures, renal function, BMI, and relevant comorbidities, and assess the robustness of their findings through prespecified sensitivity analyses and, where available, paired CSF or PET measurements. Covariate selection should reflect the research question and a plausible causal framework, since adjustment for factors downstream of genetic liability may partially obscure the association under investigation.

### 4.5. PRS Within the A/T/N Framework

In an ADNI-based study using APOE-excluded pathway PRSs, distinct pathway scores showed differential associations with amyloid, tau, and neurodegeneration-related biomarkers, according to the A/T/N AD framework [21]. In particular, amyloid positivity was linked to PRSs related to endocytosis, fibril formation, response to misfolded proteins, and tyrosine regulation [21]. By contrast, pTau positivity and hippocampal volume were linked to mitophagy and protein localization PRSs, while pTau positivity was additionally related to a PRS implicated in immune signaling [21]. These results suggest that amyloid, tau and neurodegeneration biomarkers may arise from partially overlapping but genetically distinguishable biological pressures.

Across CSF, PET, and blood-based studies, a consistent pattern emerges, according to which AD PRSs are not uniformly associated with all biomarker domains. Amyloid-related associations are generally the most reproducible, especially when APOE is included. Tau-related associations become clearer when using tau-specific, pathway-specific, or cell-type-specific PRSs. Neurodegeneration-related biomarkers, such as hippocampal volume or NfL, appear more “distal” possibly reflecting the combined effects of amyloid, tau, inflammation, vascular injury, mitochondrial dysfunction, and resilience mechanisms.

Therefore, the biomarker associations with AD PRSs may vary depending on the score composition, disease stage, and phenotype assessed. Pathway- and cell-type-specific PRSs provide a more refined approach by testing whether distinct genetic components preferentially relate to A, T, or N processes. Specific examples illustrate where non-APOE burden provides additional biological information. In BioFINDER, APOE-excluded AD PRSs were associated with CSF pTau181 and total tau in models adjusting for *APOE* ε2 and ε4 counts; selected associations persisted after additional adjustment for Aβ measures [41]. These findings support a residual association with soluble tau that is not fully accounted for by measured amyloid pathology. In Yang and colleagues, APOE-excluded cell-type-specific scores were evaluated with additional APOE adjustment: astrocytic scores were associated with amyloid pathology, whereas microglial scores were associated with tau pathology and cognitive decline, among other outcomes [24]. Thus, non-APOE information can remain relevant both within amyloid-related processes and in associations extending beyond them.

## 5. From Biomarkers to the Brain: PRSs, Brain Structure and Function

Neuroimaging represents the next stage in the genes-to-brain-to-behavior continuum by revealing how genetic susceptibility is reflected in brain structure and function. Whereas fluid and PET biomarkers reflect the molecular pathology of AD, neuroimaging studies may capture the consequences of this pathology for neural systems, at the levels of regional atrophy, cortical thinning, white matter disruption, altered network connectivity, and compensatory or resilience-related changes.

### 5.1. Structural MRI: Cortical Thickness and Regional Vulnerability

Early imaging-genetics studies showed that AD PRSs can be associated with structural differences in the brain even among cognitively normal individuals. In particular, AD PRS was correlated with decreased cortical thickness across brain regions vulnerable to AD in cognitively normal older adults, independent of *APOE* genotype and CSF Aβ42 levels [52]. Longitudinal evidence in healthy older adults demonstrated that an AD genetic risk including *APOE*, *CLU* and *PICALM* could predict greater thinning over time in the hippocampal complex region, despite no clear baseline associations, and this correlation was particularly driven by the entorhinal cortex [53]. These findings suggest that genetic susceptibility might be detectable as accelerated degeneration in brain regions vulnerable to AD pathology, being more sensitive to the time trajectories of structural brain changes than to cross-sectional differences.

Given its biological relevance to AD, the hippocampal region has received the greatest research attention. In UK Biobank, higher AD PRS was associated with smaller hippocampal volumes, with effects localized to specific subfields including the hippocampal tail, hippocampal-amygdala transition area, subiculum, CA1, CA4, molecular layer, and dentate gyrus-related regions [54]. Hence, it seems that AD PRSs may preferentially affect certain medial temporal substructures, rather than resulting in diffuse brain atrophy.

At the same time, PRS-MRI associations are generally modest and should be interpreted cautiously, as hippocampal and cortical measures are influenced by demographic, vascular, technical, and comorbid pathological factors [55,56]. Moreover, large UK Biobank data indicate that APOE effects on hippocampal volume are age-dependent, with genotype-related differences emerging mainly after age 60 [57]. Thus, PRS-MRI associations should rather be viewed as indicators of anatomical vulnerability that may become more clinically meaningful in the context of aging, molecular pathology, or reduced brain reserve.

### 5.2. From Molecular Pathology to Regional Atrophy

A key question is whether PRS-associated brain changes are independent of AD pathology or reflect the brain’s response to amyloid and tau. Sabuncu and colleagues found that AD PRS was associated with reduced cortical thickness in AD-vulnerable regions even after adjusting for *APOE* genotype and CSF Aβ42, suggesting that at least some PRS-MRI associations may not be fully explained by amyloid pathology alone [52]. It can be speculated that lower hippocampal volume in an individual with high AD PRS might reflect several overlapping processes, including earlier-life neurodevelopmental differences, lifelong vulnerability of medial temporal networks, increased sensitivity to amyloid or tau toxicity, impaired repair mechanisms, vascular or inflammatory comorbidity, or reduced reserve. Thus, neuroimaging helps elucidate where genetic susceptibility for AD is expressed in the brain, and under which conditions it becomes anatomically visible.

### 5.3. White Matter, Connectivity, and Pathway-Specific Vulnerability

Although gray matter atrophy is central to AD, white matter integrity is increasingly recognized as an important contributor to cognitive decline. As many AD-vulnerable cortical regions are involved in highly connected association networks, genetic risk may affect not only regional atrophy but also the microstructural integrity of long-range connections. Recent studies using data from the European Prevention of Alzheimer Dementia (EPAD) cohort have extended pathway-specific PRS research to white matter. In older adults without dementia, pathway-specific AD PRSs were associated with diffusion MRI measures of white matter integrity, and moderated the effects of CSF Aβ42 and pTau181 [58]. Lipid-related PRSs showed direct associations with fiber density and cross-section, whereas clearance and immune-activation PRSs modified the relationship between AD biomarkers and white matter microstructure [58]. Therefore, pathway-informed PRSs suggest that white matter vulnerability may be partly affected by AD-related biological systems, such as lipid metabolism, immune activation, clearance, and tissue homeostasis, offering a potential explanation for why individuals with similar amyloid or tau burden may differ in cognitive resilience or decline.

### 5.4. Functional Brain Networks

Functional imaging provides a complementary view of how AD genetic risk relates to brain network organization before marked atrophy is evident. The default mode network is particularly relevant because it overlaps with regions vulnerable to early amyloid deposition and AD-related metabolic disruption [59]. In a longitudinal resting-state fMRI study of cognitively normal individuals and patients with MCI, Su and colleagues found that multilocus AD genetic risk was associated with default mode network connectivity trajectories, and non-APOE genetic risk predicted functional connectivity decline among MCI participants who later converted to AD [60]. Despite the limitations of fMRI studies, including sensitivity to motion, vascular factors, preprocessing choices, and state-dependent fluctuations, these findings suggest that network dysfunction may represent an intermediate phenotype linking genetic susceptibility to later cognitive decline. However, larger biomarker-characterized studies are needed to determine how these connectivity changes relate to amyloid and tau pathology.

### 5.5. Brain Structure and Function as Mediators of Genetic Risk

Within the genes-to-brain-to-behavior framework, brain structure and function occupy a crucial mediating position. Biomarkers indicate that AD pathology is present, but neuroimaging shows how this pathology is distributed, tolerated, or amplified within neural systems. PRSs may help explain why individuals with similar amyloid or tau burden differ in hippocampal atrophy, cortical thinning, white matter integrity, network connectivity, and ultimately cognitive impairment. Future studies should combine PRSs with amyloid, tau, vascular, inflammatory, and neurodegeneration biomarkers in longitudinal designs, enabling more precise models of how genetic liability is translated into brain-level vulnerability and resilience.

## 6. From Brain to Behavior: Cognitive and Behavioral Manifestations Associated with PRSs

**Figure 4:**
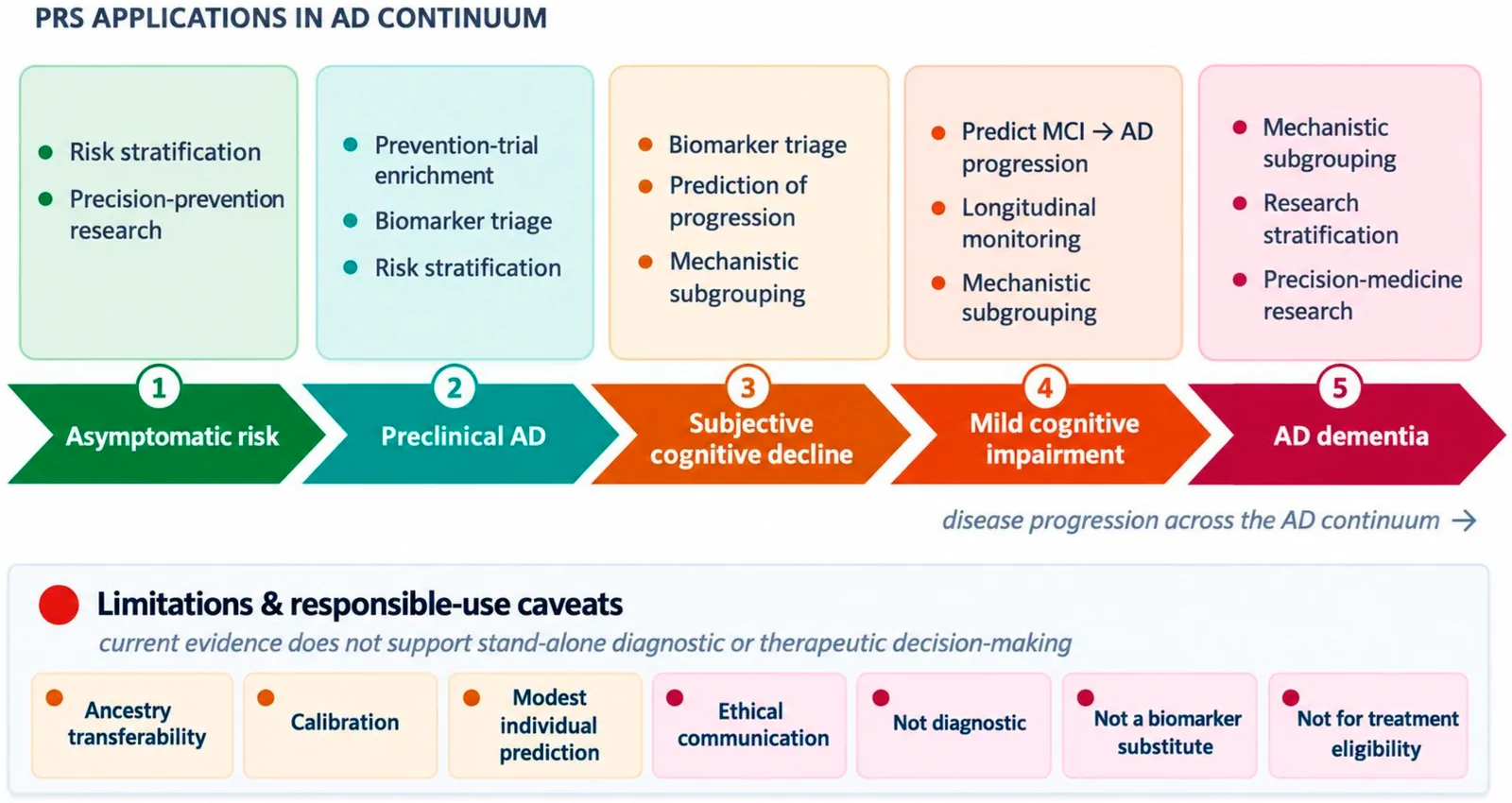
Potential applications and limitations of AD PRSs across the disease continuum. PRS may support research enrichment, biomarker prioritization, and mechanistic stratification, but current evidence does not support stand-alone diagnostic or therapeutic decision-making.

Moving beyond molecular, brain structural and functional vulnerability, PRSs might help us understand how genetic susceptibility might affect memory decline, executive dysfunction, neuropsychiatric symptoms, conversion from preclinical or prodromal stages to dementia, and resilience to pathology. However, PRS-behavior associations are generally modest and highly context dependent, being influenced by age, *APOE* status, biomarker burden, vascular comorbidities, psychiatric history, and the cognitive or behavioral phenotype under study (Figure 4).

### 6.1. Cognitive Performance in Cognitively Unimpaired Individuals

In cognitively unimpaired older adults, AD PRSs have been associated with subtle differences in cognitive performance, although findings are not fully consistent across cohorts. In the AIBL study among cognitively normal older adults with high Aβ burden, Porter and colleagues derived an episodic-memory-weighted PRS, and demonstrated that both APOE-inclusive and -exclusive memory-weighted scores were associated with longitudinal decline in global cognition and verbal episodic memory [61]. In a complementary analysis, in the EMIF-AD PreclinAD cohort, Tomassen and colleagues found that amyloid-β positivity, *APOE* genotype, and non-APOE AD PRSs each contributed to memory decline in cognitively unimpaired older adults, although APOE-related decline was most evident among amyloid-positive individuals [62]. Together, these findings suggest that cognitive prediction in preclinical AD depends on both the biological context, particularly amyloid positivity, and the way genetic risk is modeled. Therefore, it can be speculated that in preclinical AD, PRSs may modify the probability that AD molecular pathology is translated into subtle cognitive decline. Hence, cognitive outcomes can be rather conceptualized as the interaction between genetic susceptibility, AD pathology, and potentially brain integrity and functional reserve.

### 6.2. Cognitive Decline, MCI Conversion, and Dementia Progression

Associations between AD PRSs and cognition are often more informative in longitudinal compared to cross-sectional studies. Desikan and colleagues developed a polygenic hazard score to model age-associated AD risk and reported associations with clinical progression, neuropathology, and neurodegeneration markers [6]. In another study [63], combining a polygenic hazard score with volumetric MRI and cognitive measures improved prediction of progression from MCI to AD dementia. Furthermore, in participants with MCI, Pyun and colleagues reported associations of both APOE-inclusive and APOE-excluded PRSs with progression to dementia in age- and sex-adjusted models [23]. Furthermore, the interaction with *APOE* ε4 carrier status was significant for the APOE-inclusive score, but not for the APOE-excluded score.

Cognitive-domain specificity is also important. Porter and colleagues showed that an episodic-memory-weighted PRS was associated with longitudinal decline in verbal episodic memory, global cognition, and an AD-sensitive cognitive composite among cognitively normal Aβ-positive older adults [61]. In a longitudinal study among late-middle-aged individuals, Gustavson and colleagues demonstrated that AD PRS could predict cognitive decline in episodic memory and executive function, although these associations were significantly affected by the *APOE* genotype, highlighting that AD genetic liability might be expressed in several cognitive domains related to AD [64].

### 6.3. Behavioral and Neuropsychiatric Manifestations

The most direct evidence regarding the relationship between AD PRSs and neuropsychiatric symptoms (NPS) comes from studies of mild behavioral impairment (MBI), a construct developed to capture later-life emergent and persistent NPS that may signal increased risk for cognitive decline. In the PATH Through Life cohort, Andrews and colleagues showed that higher AD genetic risk and *APOE* ε4 were associated with increased likelihood of MBI affective dysregulation, while specific associations were observed between individual AD loci and other MBI domains, including abnormal perception or thought content, social inappropriateness, and decreased motivation [65]. Although exploratory, this study provided early evidence that AD genetic liability may contribute not only to cognitive vulnerability but also to specific behavioral phenotypes.

Further evidence comes from the PROTECT study, in which AD PRS was associated with poorer cognition in individuals with MBI, but not in those without MBI [66]. The association was strongest among participants older than 65 years with more severe MBI, and adjustment for APOE did not materially change the association [66]. Although a causal association has not been proven, these findings suggest that MBI may identify a subgroup in whom AD genetic liability is more clinically expressed.

Overall, current evidence suggests that AD genetic liability may contribute to behavioral phenotypes, particularly MBI and affective or motivational symptoms, but the field remains at an early stage. Future studies should combine AD PRSs with amyloid, tau, neuroinflammatory, vascular, and synucleinopathy markers. Such work will be essential to determine whether behavioral symptoms represent early AD-related clinical expression, markers of mixed pathology, or nonspecific manifestations of brain vulnerability.

### 6.4. Resilience, Reserve, and Clinical Heterogeneity

Cognitive reserve refers to the capacity to maintain better-than-expected cognitive function despite age- or disease-related brain changes, and it is commonly operationalized through education, literacy, occupational complexity, or cognitively stimulating activity. In the Preclinical AD Consortium, Pettigrew and colleagues demonstrated that *APOE* ε4 and AD PRSs including the *APOE* region were associated with decline across cognitive domains, while non-APOE AD PRS was associated with decline in executive function and global cognition [67]. In this study, the associations with executive function and global cognitive decline persisted when *APOE* ε4 status and its interaction with time were included as additional predictors, whereas the APOE-excluded score was not significantly associated with episodic memory decline. This provides evidence of domain-specific associations beyond *APOE* ε4, although the score’s incremental out-of-sample predictive performance has not been established. Importantly, higher cognitive reserve attenuated *APOE* ε4-related cognitive decline, but did not clearly attenuate decline associated with broader AD PRSs [67], suggesting that cognitive reserve may buffer some genetic risk effects, particularly those related to *APOE*.

Genetic resilience represents a complementary dimension. Whereas PRSs typically aggregate risk-increasing alleles, resilience-oriented approaches seek genetic factors that reduce penetrance among individuals who would otherwise be expected to develop disease. Polygenic resilience scores have been proposed to capture protective common-variant effects among cognitively unaffected individuals with high AD genetic risk. In this regard, the reported individual homozygous for *APOE3* Christchurch also carried the pathogenic *PSEN1* E280A mutation and showed markedly delayed clinical onset despite substantial amyloid burden, with relatively limited tau pathology and neurodegeneration [68]. Although such cases are exceptional and not directly comparable to common-variant PRSs, they illustrate that genetic background can modify the relationship between amyloid accumulation, tau spread, neurodegeneration, and clinical symptoms.

Within the genes-to-brain-to-behavior framework, reserve and resilience modify whether genetic and molecular risk becomes clinically expressed. PRS captures inherited susceptibility, biomarkers define disease biology, and imaging reflects brain vulnerability, but reserve influences the threshold for cognitive or behavioral impairment. Future studies should therefore test interactions among PRSs, AD biomarkers, neurodegeneration, vascular burden, lifestyle, and reserve to identify both risk and resilience mechanisms.

## 7. Clinical Applications of PRSs Across the AD Continuum

Current AD PRSs are probabilistic research instruments whose principal applications include risk stratification and cohort enrichment [9]. However, a high AD PRS does not establish current AD pathology or inevitable future dementia, and a low score does not exclude either. Their use to guide individual clinical decisions requires validation for the intended population and setting, together with evidence of benefit beyond established clinical and biomarker assessment.

In cognitively unimpaired individuals, PRSs may have greatest value for prevention-oriented stratification [8]. Individuals at high genetic risk may be more likely to develop amyloid positivity, show subtle biomarker abnormalities, or progress cognitively over time, particularly when PRS is combined with *APOE* [9]. In this setting, PRSs could help prioritize individuals for longitudinal monitoring, plasma biomarker testing, amyloid or tau PET, or enrollment into preclinical prevention trials [10]. In individuals with subjective cognitive decline or MCI, PRSs might contribute to prognostic stratification. Several studies have shown that AD PRSs or polygenic hazard scores can help predict progression from cognitively unimpaired states or MCI to AD dementia, particularly when combined with MRI, cognitive measures, and amyloid or tau biomarkers [9]. Furthermore, clinical trials in preclinical or prodromal AD require large samples and long follow-up, so enriching for individuals more likely to accumulate pathology or decline cognitively can improve efficiency [9]. Nevertheless, PRS-based enrichment must be implemented carefully to avoid reducing ancestry diversity or excluding individuals whose risk is driven by non-genetic, vascular, environmental, or social determinants [9].

The emergence of disease-modifying therapies further changes the clinical context in which genetic risk is interpreted [69]. Current anti-amyloid treatment pathways rely on clinical stage, biomarker confirmation of AD pathology, MRI safety screening, and *APOE* genotyping for amyloid-related imaging abnormality risk, rather than on PRSs [69]. PRSs should therefore not be used to determine treatment eligibility or to substitute for biomarker confirmation. However, in future clinical algorithms, PRSs could potentially support earlier risk triage, helping determine who may benefit from blood-based biomarker screening or referral for confirmatory testing. This role would be especially relevant if disease-modifying therapies move toward earlier symptomatic, prodromal, or eventually preclinical populations.

Pathway-specific and cell-type-specific PRSs are less immediately applicable for clinical prediction but may be valuable for mechanism-informed stratification [8]. For example, lipid-, immune-, microglial-, astrocytic-, tau-, or mitophagy-related scores may eventually help identify biological subgroups that differ in biomarker trajectories, neurodegeneration patterns, or therapeutic response. Such approaches are particularly attractive for precision medicine because of the biological heterogeneity of AD.

Despite these promising future implications, several barriers limit the clinical implementation of AD PRSs [8]. Ancestry-related transferability and calibration limitations, discussed in Section 2.3 and Section 8.1, constrain equitable clinical implementation. For these reasons, clinical use of AD PRSs should proceed cautiously. The most defensible near-term applications are in research settings, for enrichment of prevention and prodromal trials, stratification of biomarker screening studies, investigation of disease heterogeneity, and modeling of progression risk. Routine clinical deployment will require ancestry-inclusive validation, standardized reporting, clear thresholds, absolute risk calibration, prospective utility studies, and evidence that PRS-informed decisions improve patient outcomes. Until then, PRSs should be presented as one component of a broader precision medicine framework rather than as an independent clinical decision tool.

## 8. Emerging Directions

The next phase of AD PRS research is likely to move beyond global genetic risk estimation toward biologically resolved, ancestry-inclusive, longitudinal, and clinically integrated models. However, most current scores remain limited by European-ancestry discovery data, incomplete biological specificity, modest individual-level predictive performance, and insufficient prospective validation. Emerging work therefore aims not only to improve prediction, but also to clarify how different genetic architectures are expressed across the AD continuum.

### 8.1. Multi-Ancestry PRS and Equitable Implementation

Improving cross-ancestry portability requires larger and more representative discovery cohorts, ancestry-appropriate imputation and LD-reference panels, and integration of GWAS data across populations [26,27]. In admixed populations, local ancestry inference estimates the ancestral origin of individual genomic segments, complementing genome-wide ancestry estimates. Admixture mapping can test whether ancestry at a particular region is associated with disease, while local-ancestry-aware association analyses can investigate ancestry-specific variant effects. These complementary approaches can inform score development, but their contribution to predictive performance must be evaluated empirically.

Multi-ancestry methods such as PRS-CSx integrate GWAS summary statistics from multiple populations using population-specific LD reference panels and a coupled continuous shrinkage prior that shares information across populations while allowing SNP effect estimates to differ [70]. Their methodological promise should be distinguished from demonstrated clinical utility in AD. Before clinical translation, scores should undergo independent evaluation of discrimination, absolute risk calibration, and clinical net benefit in the intended populations, including admixed individuals. Evaluation should also examine whether proposed risk thresholds produce unequal false-positive or false-negative rates across groups. Equitable implementation additionally requires representative recruitment and access to genetic counseling, confirmatory assessment, and subsequent care; improved statistical portability alone cannot ensure equitable outcomes.

### 8.2. Integrating Common and Rare Genetic Variation

Future models will need to integrate common-variant PRSs with rare-variant burden. Common PRSs capture distributed population-level susceptibility, whereas rare variants in genes such as *TREM2*, *SORL1*, and *ABCA7* can exert larger effects in smaller subsets of individuals and provide stronger mechanistic insights into underlying molecular pathways [16]. Combining these layers may improve biological interpretation and risk stratification, but will require large sequencing cohorts, ancestry-aware calibration, and careful distinction between probabilistic risk alleles and pathogenic or likely pathogenic variants.

### 8.3. From Global PRS to Pathway-, Tissue-, and Cell-Type-Specific Scores

Global AD PRSs maximize aggregate prediction but obscure biological heterogeneity. Pathway-, tissue-, and cell-type-specific PRSs offer a more mechanistic approach by decomposing genetic risk into systems such as lipid metabolism, immune activation, endocytosis, mitophagy, amyloid processing, tau biology, microglial activation, astrocytic function, and neuronal vulnerability [24]. Early studies suggest that astrocytic, microglial, neuronal, and liver-informed PRSs may differentially relate to amyloid, tau, cognition, and CSF biomarker profiles [24,44]. These scores are not yet clinically actionable, but they provide a promising framework for understanding why similar global genetic risk may lead to different biomarker and clinical trajectories.

### 8.4. Multi-Omics and Functional Annotation

Functional annotation can improve the biological interpretation of AD PRSs by linking associated variants to candidate effector genes and relevant cellular contexts. Variant-to-gene mapping should integrate complementary evidence. Expression quantitative trait loci (eQTLs), splicing QTLs (sQTLs), and protein QTLs (pQTLs) connect genetic variation to transcript abundance, RNA processing, and protein abundance, respectively. Colocalization analyses assess whether GWAS and molecular QTL associations are consistent with a shared causal signal, although further research is needed to elucidate whether the molecular phenotype mediates disease risk. Bellenguez and colleagues illustrate this integrative approach through gene prioritization incorporating molecular QTL evidence, including brain pQTLs, and colocalization analyses of expression and splicing signals [15].

Chromatin accessibility profiles, such as those generated by ATAC-seq, identify candidate regulatory regions, while enhancer–promoter interaction maps help connect distal regulatory elements to potential target genes. These approaches can provide information that is not captured by assigning variants to the nearest gene. For example, Nott and colleagues mapped regulatory interactions in human brain cell types and demonstrated that deletion of an AD-risk-associated enhancer markedly reduced BIN1 expression in stem-cell-derived microglia, without a comparable effect in neurons or astrocytes [71]. This supports a cell-dependent regulatory mechanism, but does not establish that therapeutic modulation of BIN1 would benefit patients.

Functional mapping should also be distinguished from the annotation rules used to construct cell-type-specific PRSs. Yang and colleagues, for example, selected genes using single-nucleus expression specificity and calculated scores from variants within those genes and their flanking regions [24]. Although such scores localize aggregate genetic associations to annotated gene sets, they do not indicate that every included variant acts exclusively in the designated cell type. Future annotation-informed scores should therefore assess sensitivity to gene-mapping rules, shared genes across cell types, and the tissue and disease-state context of the reference datasets.

Comparative expression datasets may provide an additional annotation layer by identifying conserved mitochondrial maintenance programs for investigation [34,35]. Their application to AD PRSs should remain hypothesis-driven: conservation or tissue enrichment alone is insufficient to classify a gene as an AD susceptibility gene or assign a variant a causal effect. Candidate pathways involving RNA surveillance, oxidative protein repair, and regulated transport should be evaluated against human AD genetic associations, relevant molecular QTLs, and cell-resolved functional evidence before incorporation into mechanistically interpreted scores.

For drug discovery, convergence of genetic association, molecular annotation, and functional perturbation can prioritize candidate targets and the cellular systems in which to investigate them. However, a high pathway- or cell-type-specific PRS does not by itself indicate whether a target should be activated or inhibited. For clinical trials, these scores could be investigated alongside biomarkers and clinical staging for biological subgrouping or cohort enrichment. Importantly, prediction of treatment response should be evaluated through prespecified treatment-by-score interaction analyses in adequately powered randomized trials. Prospective validation is therefore needed before using these scores to determine trial eligibility or treatment selection.

### 8.5. Biomarker-Specific and Endophenotype-Derived PRS

A key emerging direction is the development of PRSs derived from biomarkers or AD endophenotypes. Tau PET-derived PRSs, plasma biomarker GWAS, neuroimaging-derived scores, and amyloid- or tau-specific genetic models may better capture the genetic architecture of particular disease processes [46,72]. This approach could help distinguish inherited susceptibility to amyloid accumulation, tau spread, glial activation, neuroaxonal injury, or rapid progression [72]. In the future, disease risk PRSs may be integrated with biomarker-specific genetic scores to model not only AD dementia risk, but also the molecular route through which that risk is expressed.

### 8.6. Longitudinal and Causal Modeling Across the AD Continuum

Because AD develops over decades, longitudinal designs are essential for determining when PRS effects become visible and whether they influence biomarker initiation, progression rate, neurodegeneration, or clinical conversion [6]. Repeated CSF, MRI, and cognitive measures will allow genetic risk to be modeled dynamically rather than cross-sectionally [6]. Mediation, joint longitudinal modeling, disease-progression modeling, and causal inference approaches may help clarify whether PRS effects on cognition or behavior are mediated through amyloid, tau, glial activation, vascular injury, brain atrophy, or reserve. Mendelian randomization, which uses genetic variants as instrumental variables to test whether an exposure is causally rather than merely correlationally related to an outcome, is particularly suited to this task. Combining PRSs with two-sample Mendelian randomization across 22 candidate risk factors and the AD phenome, including age at onset, hippocampal volume, cortical thickness, CSF biomarkers, and neuropathological burden, has already shown that several observationally associated exposures are not supported as causal [73].

### 8.7. Multimodal Prediction and Artificial Intelligence

PRSs are unlikely to be clinically useful as stand-alone predictors, but they may add value within multimodal models that combine age, sex, education, family history, APOE, blood biomarkers, amyloid and tau measures, MRI, vascular risk, lifestyle, digital cognition, and clinical symptoms [63]. Machine-learning approaches may help integrate these data for trial enrichment or individualized prognosis, but they must demonstrate calibration, interpretability, fairness, and transportability [63]. The critical question is not whether PRS is statistically associated with AD outcomes, but whether it improves prediction beyond established markers such as age, APOE, plasma pTau217, amyloid status, MRI, and baseline cognition.

### 8.8. PRS in the Era of Disease-Modifying Therapy

Beyond the current clinical requirements outlined in Section 7, an unresolved question is whether AD PRSs can improve recruitment efficiency or identify heterogeneity in treatment response. These possibilities should be evaluated prospectively, distinguishing enrichment for disease risk or progression from prediction of differential treatment benefit [69].

### 8.9. Ethical, Regulatory, and Communication Challenges

Clinical translation of AD PRSs raises substantial ethical and regulatory challenges. PRS results are probabilistic, ancestry-dependent, and easily misunderstood as deterministic, particularly in a disease associated with psychological distress and family implications. Implementation will require standards for absolute risk calibration, privacy, reporting thresholds, and protection against inequitable use [28]. PRSs should not be used to restrict access to care or falsely reassure individuals at lower genetic risk, since dementia risk also depends on age, vascular disease, environment, lifestyle, and non-AD pathologies.

Where individual PRS results are returned, disclosure should follow an explicit informed-consent process that addresses the option not to receive results. Pre- and post-disclosure counseling should explain the distinction between inherited susceptibility, biomarker evidence of pathology, and a clinical diagnosis. Communication should identify the score’s reference population, whether APOE is included, and the limitations of individual prediction. Estimates should be presented only when supported by an appropriately validated model, with the relevant time horizon and uncertainty, and a percentile should not be presented as a disease probability. Disclosure procedures should address possible psychological distress, implications for relatives, confidentiality, and potential insurance or employment consequences in the applicable jurisdiction. Research participation should not automatically imply consent to receive genetic risk information, and participants should have access to follow-up support and clarification.

### 8.10. Toward a Cross-Scale Precision Medicine Framework

The future of AD PRSs lies in cross-scale integration. Global PRSs can estimate aggregate inherited susceptibility; pathway-, tissue-, and cell-type-specific PRSs can clarify biological routes; biomarker-specific PRSs can capture molecular-stage risk; and multimodal models can place genetic risk within the broader context of biomarkers, brain vulnerability, cognition, behavior, lifestyle, and comorbidity [8,37,46,63]. The goal is not simply to improve prediction, but to understand why genetic risk is expressed differently across individuals and to identify mechanisms through which susceptibility can be delayed, modified, or prevented.

## 9. Conclusions

AD PRSs have substantially expanded the way genetic susceptibility is studied across the AD continuum. Rather than representing simple predictors of future dementia, PRSs provide a framework for understanding how inherited risk is distributed across biological pathways, molecular biomarkers, brain systems, cognitive trajectories, and behavioral manifestations. This cross-scale perspective is essential because AD genetic liability is not expressed uniformly. The same aggregate genetic risk may manifest through amyloid accumulation, tau dysregulation, glial activation, white matter vulnerability, hippocampal atrophy, cognitive decline, neuropsychiatric symptoms, or resilience, depending on *APOE* status, disease stage, biomarker burden, ancestry, comorbidity, environmental exposures, and reserve.

Current evidence suggests that conventional AD PRSs are most robustly associated with amyloid-related processes, particularly when APOE is included. Associations with tau, neurodegeneration, brain structure, functional networks, cognition, and behavior are more variable, but become more informative when PRSs are modeled longitudinally or refined through pathway-, cell-type-, tissue-, or biomarker-specific approaches. These emerging models suggest that AD genetic risk is biologically heterogeneous and may act through partly distinct mechanisms, including lipid metabolism, endosomal trafficking, immune activation, microglial and astrocytic function, proteostasis, and mitochondrial quality control. Such approaches are especially valuable for explaining why individuals with similar amyloid or tau burden may differ in brain atrophy, cognitive impairment, behavioral symptoms, and clinical progression.

Despite this promise, PRSs are not yet ready for routine stand-alone clinical use. Their predictive value at the individual level remains modest, their transferability across ancestries is incomplete, and their interpretation requires careful calibration against age, APOE, biomarkers, cognition, neuroimaging, vascular risk, lifestyle, and family history. At present, the most defensible applications are in research settings, including trial enrichment, biomarker stratification, progression modeling, and mechanistic subgrouping. Future work should prioritize ancestry-inclusive GWAS, integration of common and rare variants, multi-omic functional annotation, longitudinal biomarker-rich cohorts, and ethically responsible implementation.

Overall, PRSs should be conceptualized as general probabilistic measures of inherited susceptibility whose clinical meaning depends on downstream biological and neural context. Their greatest value lies not in replacing biomarkers or clinical assessment, but in helping connect genomic variation to the molecular, brain-level, cognitive, and behavioral heterogeneity of AD. Within a precision medicine framework, PRSs may ultimately contribute to earlier risk stratification, more efficient clinical trials, and a deeper mechanistic understanding of why AD develops, progresses, and manifests differently across individuals.

## Figures and Tables

**Table 1:** Methodological characteristics and reported outcomes of representative AD polygenic-score studies.

Study	Discovery GWAS	Target Cohort and Sample Size (N)	Ancestry of Target Sample	PRS Method and Variant-Selection Threshold	APOE Handling	Outcomes	Reported Performance or Association
[5]	IGAP 2013 stage 1: 17,008 cases and 37,154 controls.	Neuropathologically characterized cohort: 1011 cases and 583 controls.	Not explicitly specified in the 2017 report; cohort characteristics referenced to the original dataset publication.	Effect-size-weighted PRS using LD-pruned SNPs; *p*-value thresholds from 10^−4^ to 0.5 were evaluated. The best-performing threshold was *p* ≤ 0.5 (205,068 SNPs)	APOE signal included directly or through proxy variants.	Discrimination between neuropathologically confirmed AD cases and controls.	Observed AUC: 0.866. Simulation-adjusted AUC accounting for discovery–target overlap: 0.84 (95% CI 0.82–0.86).
[6]	IGAP 2013 for initial variant selection; ADGC phase 1 for estimation of polygenic hazard-score weights.	Development: 6409 cases and 9386 controls. Replication: 20,680 participants across ADGC phase 2, NIA ADC, and ADNI datasets.	Predominantly European ancestry; non-European ancestry excluded from the ADGC analyses.	Polygenic hazard score (PHS) constructed using stepwise Cox proportional hazards modeling. Candidate SNPs were initially selected at GWAS *p* < 10^−5^; the final model included 31 SNPs plus two APOE variants and incorporated population-based age-specific AD incidence rates.	APOE included; additional analyses restricted to APOE ε3/ε3 participants.	Age at AD onset and age-specific AD hazard; additional clinical and neuropathological validation outcomes.	Development cohort, APOE ε3/ε3 subgroup: highest versus lowest PHS decile, HR 3.34 (95% CI 2.62–4.24). In ADGC Phase 2 AD cases, predicted age at onset correlated with mean observed age at onset across percentile bins (r = 0.90).
[23]	Jansen et al., 2019 AD GWAS [14].	732 ADNI participants with MCI; 270 progressed to dementia within five years.	Non-Hispanic participants of European ancestry, selected using genetic ancestry analysis.	Clumping and thresholding using PRSice. Seven *p*-value thresholds were evaluated (10^−6^, 10^−5^, 10^−4^, 10^−3^, 10^−2^, 0.05, and 0.5); *p* < 10^−5^ was selected based on performance in the target sample.	Both APOE-inclusive and APOE-exclusive scores. Exclusion interval extended from 1 Mb upstream of rs429358 to 1 Mb downstream of rs7412.	Progression from MCI to dementia during up to 5 years of follow-up.	Age- and sex-adjusted HR per score z-unit: 1.468 (95% CI 1.335–1.615) for the inclusive score and 1.293 (95% CI 1.157–1.445) for the exclusive score. Threshold selection within the target cohort limits inference about external predictive performance.
[24]	Bellenguez et al. stage I AD GWAS [15].	ROSMAP autopsy cohort: 1457 participants; independent A4 screening dataset: 2921 cognitively unimpaired participants, with outcome-specific subsets.	European ancestry; A4 analyses restricted to non-Hispanic White participants after exclusion of genetic ancestry outliers.	Bayesian continuous shrinkage using PRS-CS-auto, without a GWAS *p*-value inclusion threshold. Cell-type-specific scores used posterior SNP weights for variants within genes in the top 10% of cell-type expression specificity, including ±30 kb flanking regions.	APOE region excluded (APOE ± 1 Mb); models additionally adjusted for APOE ε2 and ε4 allele counts.	AD dementia status and AD-related endophenotypes, including amyloid and tau pathology, neurodegeneration, and cognition; outcomes differed between ROSMAP and A4.	Incremental model fit was assessed using ΔNagelkerke’s R^2^ for AD dementia case–control status and Δadjusted R^2^ for quantitative endophenotypes. In ROSMAP, PRS-CS-based scores yielded larger ΔR^2^ than PRSet scores in 14 of 15 selected significant cell-type-specific PRS–endophenotype associations; 10 comparisons had empirical bootstrap *p* < 0.05. These are outcome-specific model-fit measures, not externally validated diagnostic discrimination. The authors reported that each cell-type-specific score explained 3% or less of the variance in each AD endophenotype.

## Data Availability

No new data were created or analyzed in this study. Data sharing is not applicable to this article.

## Abbreviations

The following abbreviations are used in this manuscript:

Aβ	amyloid-β
AD	Alzheimer’s disease
ADGC	Alzheimer’s disease genetics consortium
ADNI	Alzheimer’s disease neuroimaging initiative
ADRD	AD-related dementias
APP	amyloid precursor protein
AUC	area under the receiver operating characteristic curve
CI	confidence interval
CSF	cerebrospinal fluid
eGFR	estimated glomerular filtration rate
EOAD	early-onset AD
eQTL	expression quantitative trait loci
GFAP	glial fibrillary acidic protein
GWAS	genome-wide association studies
HR	hazard ratio
IGAP	International genomics of Alzheimer’s project
LD	linkage disequilibrium
LOAD	late-onset AD
LUBAC	the linear ubiquitin chain assembly complex
MBI	mild behavioral impairment
MCI	mild cognitive impairment
NfL	neurofilament light
NFT	neurofibrillary tangles
NIA ADC	national institute on aging Alzheimer’s disease centers
NPS	neuropsychiatric symptoms
PD	Parkinson’s disease
PET	positron emission tomography
pQTL	protein quantitative trait loci
PRS	polygenic risk score
ROSMAP	religious orders study and rush memory and aging project
SNP	single nucleotide polymorphism
